# The Safety of Minimally Invasive and Open Cholecystectomy in Elderly Patients With Acute Cholecystitis: A Systematic Review

**DOI:** 10.7759/cureus.31170

**Published:** 2022-11-06

**Authors:** Diana M Montenegro, Michael Chukwu, Paghunda Ehsan, Rawia N Aburumman, Shivani Ishwarya Muthanna, Swathi Radhakrishnan Menon, Vruti Vithani, Bansi Sutariya, Ann Kashmer Yu

**Affiliations:** 1 General Surgery, California Institute of Behavioral Neurosciences & Psychology, Fairfield, USA; 2 Emergency Department, Pilgrim Hospital Boston, Boston, GBR; 3 Research, California Institute of Behavioral Neurosciences & Psychology, Fairfield, USA; 4 Internal Medicine, Hayatabad Medical Complex, Peshawar, PAK; 5 Internal Medicine, Lady Reading Hospital, Peshawar, PAK; 6 Internal Medicine, California Institute of Behavioral Neurosciences & Psychology, Fairfield, USA; 7 General Medicine, Mu’tah University, Amman, JOR; 8 Pediatrics, California Institute of Behavioral Neurosciences & Psychology, Fairfield, USA

**Keywords:** elderly patient, acute cholecystitis, open cholecystectomy, laparoscopic cholecystectomy, minimally invasive cholecystectomy

## Abstract

Elderly patients with acute cholecystitis (AC) often receive no surgical treatment due to a high number of comorbidities and a high risk of operations. With an increasingly aged population worldwide, this systematic review aims to review the safety of minimally invasive cholecystectomy and open cholecystectomy in this population compared to younger patients. A systematic search was conducted on PubMed, PubMed Central, and Google Scholar databases on July 2, 2022. Articles in the English language published in the last five years with free full text and involving elderly patients with AC treated with minimally invasive and open cholecystectomy were selected. Moreover, a quality assessment was carried out by using each study's most commonly used assessment tools.

Initially, the search yielded 1,252 potentially relevant articles. After the final selection process, 11 studies were included: one cross-sectional study, eight cohort studies, one case-control study, and one systematic review with meta-analyses. These studies involved a total of 378,986 participants, with 375,623 elderly patients. In the elderly, cholecystitis severity, decreased physical status, and multiple comorbidities increase the risk of complications with cholecystectomy. In addition, the elderly had more complications, open surgery conversions, biliary tract injuries, leaks, postoperative mortality, and hospital length of stay than younger patients. Nevertheless, minimally invasive cholecystectomy is a viable treatment option for elderly patients when performing a thorough perioperative assessment.

## Introduction and background

According to the 2020 Profile of Older Americans by the Administration for Community Living (ACL), the population older than 65 years accounted for 54.1 million people in 2019, representing around 16% of the population in the United States [1]. As the population ages, there is a rise in the incidence, severity, and complications of gallstone diseases, along with an increase in surgical demands [2]. The specific characteristics of older patients increase the risk of complications due to surgical therapies. In addition, the heterogeneity of this group with regard to comorbidities and their diminished physical capabilities decrease their capacity to adapt and make them more susceptible to adverse outcomes [3].

Acute cholecystitis (AC) results from the obstruction of the cystic duct, typically by a gallstone, followed by gallbladder distension and bacterial or chemical inflammation. The typical symptoms of AC include right upper quadrant pain, anorexia, nausea, vomiting, and fever [4,5]. AC usually requires rapid treatment due to the risk of potentially severe complications if left untreated. The only permanent cure for symptomatic gallstone disease is cholecystectomy. The Tokyo guidelines recommend early cholecystectomy as an adequate treatment [5].

Cholecystectomy using laparoscopy (LC) has been the operative standard of care since the late 1990s [6], and an overall reduction in operative mortality per procedure has been observed when compared with open cholecystectomy [7,8]. However, it is still debated whether cholecystectomy should be used as the first line of treatment for AC in elderly patients. In light of this, this systematic review aims to evaluate the safety of the different surgical treatments for AC in elderly patients (>65 years) compared to younger patients.

## Review

Methods

This systematic review was conducted according to the Preferred Reporting Items for Systematic Reviews and Meta-Analyses (PRISMA) 2020 guideline [9].

Inclusion and Exclusion Criteria

The studies were selected based on the participant, the intervention, and the outcome (PIO) element: participants - elderly patients with AC; intervention - laparoscopic cholecystectomy, robotic-assisted cholecystectomy, or primarily open cholecystectomy; and outcome - results of the surgery and any complication reported. Other inclusion criteria were English language articles published in the last five years with free full text available. The exclusion criteria were conference papers, abstracts, guidelines, case reports, and any studies that did not involve AC.

Search Strategy

A systematic search of the current literature was conducted on PubMed, PubMed Central (PMC), and Google Scholar databases by two independent authors; the last day of inquiry on all the databases was July 2, 2022. The search terms used were 'Minimally invasive cholecystectomy' OR 'Laparoscopic cholecystectomy' OR 'robotic-assisted cholecystectomy' and 'Open cholecystectomy' and 'Acute cholecystitis' and 'Elderly patient' individually or in combination. All the search terms used for this review are shown in Table 1.

**Table 1:** The strategy for conducting bibliographic searches in databases and the corresponding filters PMC: PubMed Central; MeSH: Medical Subject Headings; Majr: the major topic of an article

Databases	Keywords	Search strategy	Filters	Search results
PubMed	Minimally invasive cholecystectomy, Laparoscopic cholecystectomy, Robotic-assisted cholecystectomy, Open cholecystectomy, Acute cholecystitis, Elderly patients.	#1Minimal invasive cholecystectomy OR Laparoscopic cholecystectomy OR Robotic-assisted cholecystectomy OR ("Cholecystectomy, Laparoscopic/methods"[MeSH] OR "Cholecystectomy, Laparoscopic/standards"[MeSH] OR "Cholecystectomy, Laparoscopic/therapeutic use"[MeSH]) #2Open Cholecystectomy OR Open gallbladder surgical excision OR Open gallbladder removal OR Open gallbladder surgery OR ("Cholecystectomy/complications"[MeSH] OR "Cholecystectomy/methods"[MeSH] OR "Cholecystectomy/standards"[MeSH] OR "Cholecystectomy/therapeutic use"[MeSH]). #3Acute cholecystitis OR Gallbladder inflammation OR ("Cholecystitis, Acute/classification"[MeSH] OR "Cholecystitis, Acute/surgery"[MeSH] OR "Cholecystitis, Acute/therapy"[MeSH]) #4 Elderly patient OR Elderly person OR Geriatric patient OR "Aged/surgery"[Majr] #5(((#1) AND (#3)) AND (#3)) AND (#4)	Last five years, English language, Free full text.	251
PMC	Minimally invasive cholecystectomy, Laparoscopic cholecystectomy, Robotic-assisted cholecystectomy, Open cholecystectomy, Acute cholecystitis, Elderly patients.	#1Minimal invasive cholecystectomy OR Laparoscopic cholecystectomy OR Robotic-assisted cholecystectomy #2 Open cholecystectomy OR Open gallbladder surgical excision OR Open gallbladder removal OR Open gallbladder surgery #3 Acute cholecystitis OR Gallbladder inflammation OR ("Cholecystitis, Acute/classification"[MeSH] #4 Elderly patient OR Elderly person OR Geriatric patient OR "Aged/surgery"[Majr] #5 (#1) AND (#2) AND (#3) AND (#4)	Last five years, English language, Free full text	923
Google Scholar	Minimally invasive cholecystectomy, Laparoscopic cholecystectomy, Robotic-assisted cholecystectomy, Open cholecystectomy, Acute cholecystitis, Elderly patients.	"Minimally invasive cholecystectomy" AND "Laparoscopic cholecystectomy" OR "Robotic-assisted cholecystectomy" AND "Open cholecystectomy" AND "Acute cholecystitis" AND "Elderly patient."	2018-2022. Review articles	78

The references of all the articles were grouped and organized in alphabetic order using Excel 2021. Then, two authors removed the duplicates, they also reviewed the titles and abstracts independently and excluded any irrelevant articles. Then the complete articles of the studies identified were retrieved and reviewed. The investigators decided to exclude conference papers, abstracts, guidelines, and case reports due to the lack of analysis required for this study. They included the sole systematic review conducted in the field so far.

Risk of Bias in Individual Studies

The remaining full articles were assessed by two independents authors for quality assessment and risk bias using different tools depending on the type of study: cross-sectional studies, Joanna Briggs Institute (JBI) critical appraisal checklist; cohort and case-control studies, the Newcastle-Ottawa Scale (NOS); systematic review and meta-analyses, assessment of multiple systematic reviews 2 (AMSTAR 2) [10-12]. The assessment tools had their criteria and different scoring. When the tool scores "YES," "PARTIAL YES," or "1," a point is given. When "2" is indicated, two points are given. A minimum of 70% score for each assessment tool was accepted (Table 2).

**Table 2:** Evaluation of the quality of each type of study JBI: Joanna Briggs Institute; NOS: Newcastle-Ottawa Scale; AMSTAR 2: assessment of multiple systematic reviews 2; RoB: risk of bias

Quality assessment tool	Type of Study	Items and their characteristics	Total score	Accepted score (>70%)	Accepted studies
JBI	Cross-sectional	Eight items: (1) Were the criteria for inclusion in the sample clearly defined? (2) Were the study subjects and the setting described in detail? (3) Was the exposure measured validly and reliably? (4) Were objective, standard criteria used to measure the condition? (5) Were confounding factors identified? (6) Were strategies to deal with confounding factors stated? (7) Were the outcomes measured validly and reliably? (8) Was appropriate statistical analysis used?	8	6	Bass et al., 2021 [13]
NOS	Cohort	Eight items: (1) Representativeness of the exposed cohort. (2) Selection of the non-exposed cohort. (3) Ascertainment of exposure. (4) Demonstration that outcome of interest was not present at the start of the study. (5) Comparability of cohorts based on the design or analysis*. (6). Assessment of outcome. (7) Was follow-up long enough for outcomes to occur? (8) Adequacy of follow-up of cohorts. Scoring was done by placing a point on each category. Scored as 0, 1, 2. *Maximum of two points are allotted in this category	9	7	Wiggins et al., 2018 [14] Ekici et al., 2018 [15] Loozen et al., 2018 [16] Shin et al., 2018 [17] Escartín et al., 2019 [18] Oldani et al. 2019 [19] Coelho et al., 2020 [20] Serban et al., 2021 [21]
NOS	Case-control	Eight items: (1) Is the case definition adequate? (2) Representativeness of the cases. (3) Selection of controls. (4) Definition of controls. (5) Comparability of cases and controls based on the design and analysis*. (6) Ascertainment of exposure. (7) The same method of ascertainment for cases and controls. (8) No response rate. Scoring was done by placing a point on each category. Scored as 0, 1, 2. *Maximum of two points are allotted in this category	9	7	Xu et al., 2022 [22]
AMSTAR	A systematic review and meta-analysis	Sixteen items: (1) Did the research questions and inclusion criteria for the review include the components of PICO? (2) Did the review report explicitly state that the review methods were established before the conduct, and did the report justify any significant deviations from the protocol? (3) Did the review authors explain their selection of the study designs for inclusion in the review? (4) Did the review authors use a comprehensive literature search strategy? (5) Did the review authors perform study selection in duplicate? (6) Did the review authors perform data extraction in duplicate? (7) Did the review authors provide a list of excluded studies and justify the exclusions? (8) Did the authors describe the included studies adequately? (9) Did the review authors use a satisfactory technique for assessing the risk of bias (RoB) in individual studies included in the review? (10) Did the review authors report on the sources of funding for the studies included in the review? (11) If meta-analysis was justified, did the review authors use appropriate methods for the statistical combination of results? (12) If a meta-analysis was performed, did the review authors assess the potential impact of RoB in individual studies on the results of the meta-analysis or other evidence synthesis? (13) Did the review authors account for RoB in individual studies when interpreting/discussing the review results? (14) Did the review authors provide a satisfactory explanation for and discussion of any heterogeneity observed in the review results? (15) If they performed quantitative synthesis, did the review authors carry out an adequate investigation of publication bias (small study bias) and discuss its likely impact on the results of the review? (16) Did the review authors report any potential sources of conflict of interest, including any funding they received for conducting the review? Scored as YES or NO. A partial Yes was considered as a point	16	12	Kamarajah et al., 2020 [23]

Data Collection and Analysis

Two authors extracted the data independently. Due to the varying measures of observer variability between the studies, such as heterogeneity of participants, intervention, and outcome measures, this systematic review describes these studies based on their outcomes in a narrative synthesis. Complete articles were analyzed and tabulated into a table. The data collected for each study include first author, year of publication, study type, country of origin, number of patients, the definition of elderly or age ranges, indications of surgery, type of surgery, preoperative evaluation of the anesthetic-surgical risk based on the American Society of Anesthesiology Physical Status (ASA PS), comorbidity, and the results.

Study Outcome

The outcomes analyzed were the overall complications reported, the rate of conversion to open surgery, bile leaks and biliary tract injury, postoperative mortality, and hospital stay duration.

Results

Evaluation of Study Selection and Quality

**Figure 1:**
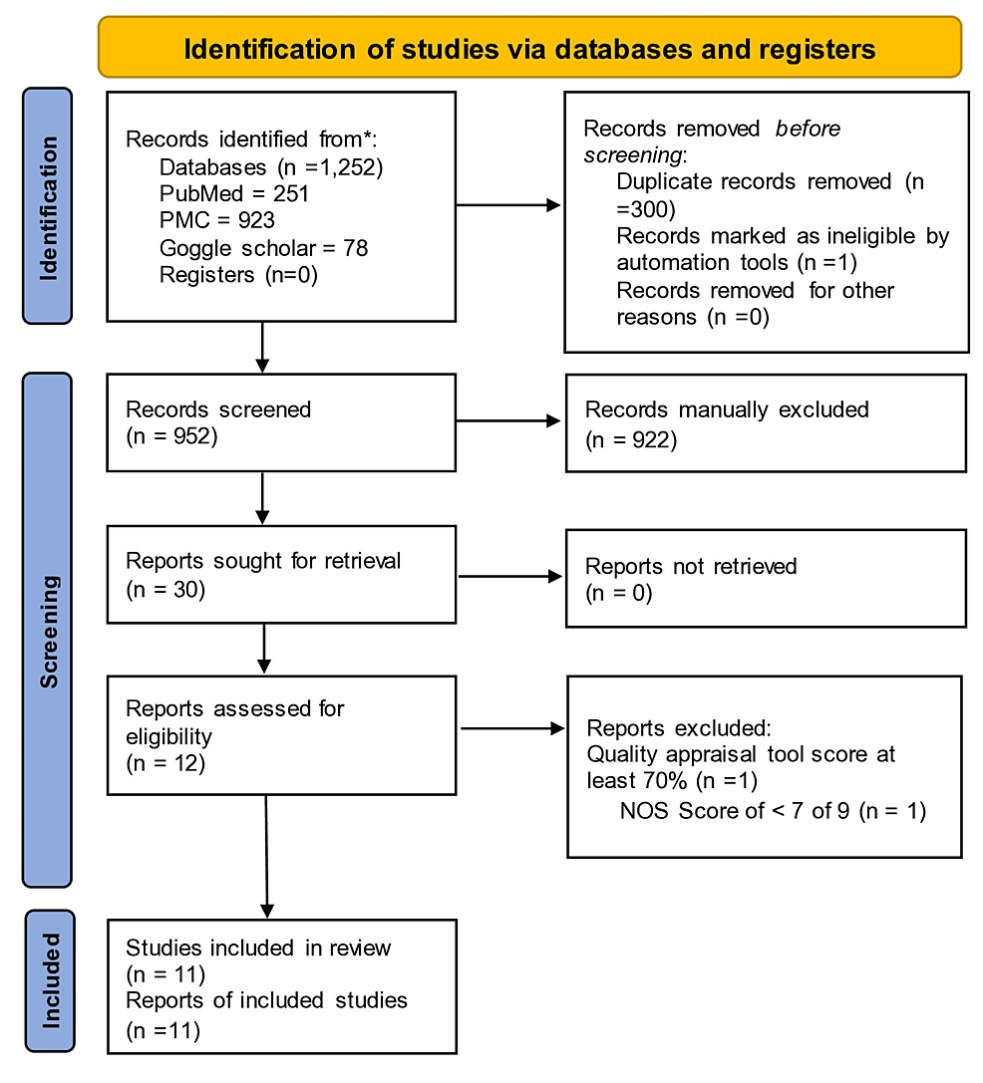
PRISMA flow chart depicting the study selection process PMC: PubMed Central; NOS: Newcastle-Ottawa Scale; PRISMA: Preferred Reporting Items for Systematic Reviews and Meta-analyses

The database search yielded a total of 1,252 potentially relevant titles. Google Scholar automatically deleted one title. The removal of duplicates was also done, with 952 records retained. A total of 30 articles remained when the titles and abstracts of these records were screened based on this review's PIO elements and eligibility criteria; these articles were retrieved, and conference papers, abstracts, guidelines, and case reports were excluded (18 articles). Finally, the quality assessment for each article was done, and 11 studies with a score of greater than 70% were accepted for the review. These included one cross-sectional study, eight cohort studies, one case-control study, and one systematic review with meta-analyses. No other resources were added. We followed the PRISMA 2020 guidelines for screening and study selection. Figure 1 shows the PRISMA flow diagram illustrating the process [9].

Each study was evaluated with the appropriate quality assessment tool for each study type, and the results were tabulated. For example, the only cross-sectional study in the review was assessed using the JBI tool. This study scored 7/8, with item 6 recorded as "NO" because the strategies to deal with confounding factors were not stated. This information is shown in Table 3 below.

**Table 3:** Summary of the critical appraisal results for the cross-sectional study by review authors Y: yes; N: no

First author, year	Item 1	Item 2	Item 3	Item 4	Item 5	Item 6	Item 7	Item 8
Bass et al. 2021 [13]	Y	Y	Y	Y	Y	N	Y	Y

NOS tool was used in assessing all the cohort studies, and most of the accepted cohort studies had a score of "1" for each item; two studies used multiple controls for the confounding factor in the analysis scoring "2" in item 5. Most studies fail to assess the adequacy of the follow-ups, scoring 0 in item 8. One study that scored 6 (<70% of quality) was excluded from the review. The summary of the assessment is presented in Table 4.

**Table 4:** Review authors' summary of the coding manual for cohort studies

First author, year	Item 1	Item 2	Item 3	Item 4	Item 5	Item 6	Item 7	Item 8
Wiggins et al., 2018 [14]	0	1	1	1	2	1	1	1
Ekici et al., 2018 [15]	1	1	1	1	1	1	1	0
Loozen et al., 2018 [16]	1	1	1	1	1	1	1	0
Shin et al., 2018 [17]	1	1	1	1	1	1	1	0
Escartín et al., 2019 [18]	0	1	1	1	1	1	1	1
Oldani et al., 2019 [19]	1	1	1	1	1	1	1	0
Coelho et al., 2020 [20]	1	1	1	1	1	1	1	0
Kim et al., 2021 [24]	0	1	1	1	1	1	1	0
Serban et al., 2021 [21]	1	1	1	1	2	1	1	0

The case-control study was assessed using the NOS tool, and the accepted study had a score of "1" for each item, with a total of 8/9 (Table 5).

**Table 5:** Result summary of coding manual for the case-control study by review authors

First author, year	Item 1	Item 2	Item 3	Item 4	Item 5	Item 6	Item 7	Item 8
Xu et al., 2022 [22]	1	1	1	1	1	1	1	1

One study was a systematic review with meta-analysis. Upon scoring using AMSTAR 2 tool, the accepted reviews had "NO" in items 3 and "Partial Yes" in items 4 and 8. These items discussed the explanation of the selection of the study designs, literature search, and studies description, respectively, as presented in Table 6.

**Table 6:** Summary of the critical appraisal for the systematic review and meta-analysis by review authors Y: yes; PY: partial yes; N: no

First author, year	Item 1	Item 2	Item 3	Item 4	Item 5	Item 6	Item 7	Item 8	Item 9	Item 10	Item 11	Item 12	Item 13	Item 14	Item 15	Item 16
Kamarajah et al., 2020 [23]	Y	Y	N	PY	Y	Y	Y	PY	Y	Y	Y	Y	Y	Y	Y	Y

Study Characteristics

The key traits of the cohorts, case-control study, cross-sectional analyses, and the systematic review with meta-analysis in the review are presented in chronological order in Tables 7-8, respectively. The studies included 378,986 participants, with 44,655 receiving conservative treatment alone, 334,239 receiving laparoscopic cholecystectomy, 92 receiving primary open cholecystectomy, and 59,350 having to be converted to open cholecystectomy. Four of the 11 studies included in the review focused on treating elderly patients, and seven compared treatments for young and elderly patients.

**Table 7:** Main characteristics of the cohort studies included in the review NR: not reported; AC: acute cholecystitis; LC: laparoscopic cholecystectomy; OC: open cholecystectomy; ASA PS: American Society of Anesthesiologists Physical Status Classification; CCI: Charlson Comorbidity Index

First author, year	Country of origin	Patient number	Age ranges	Indications for surgery	Type of treatment	ASA PS	Comorbidity	Results and key points
Wiggins et al., 2018 [14]	United Kingdom	47,500	Elderly patients >80 years	Acute cholecystitis admitted through the emergency department	Conservative treatment (89.7%, n=42,620) Cholecystectomy (7.5%, n=3,539) with a laparoscopic approach rate of 59%. Percutaneous Cholecystostomy (2.8%, n=1,341)	NR	Significantly fewer comorbidities were present in patients who underwent emergency cholecystectomy (CCI below 2 in 87.5% of patients in this group vs. 83.1% in the conservative group and 83.2% in the percutaneous cholecystostomy group)	Reduced readmission rates and 1-year mortality are two potential advantages of emergency cholecystectomy in very elderly patients. Compared to open surgery, LC had a relative risk reduction in 30-day mortality of 84%
Ekici et al., 2018 [15]	Turkey	665	Two groups: >60 years of age (127) and <60 years of age (538)	Sixty-four (9.6%) patients underwent surgery when they were experiencing AC, and 117 (17.6%) patients underwent surgery eight weeks after receiving medical care	- LC: 115 patients >60 years of age, and 513 <60 years of age	ASA scores for most patients were I: 463 (69.6%) and II: 184 (27.7%)	Hypertension 110 (16.5%), diabetes mellitus 61 (9.2%), chronic obstructive pulmonary disease 53 (8%), and congestive heart failure (4.1%) were the most prevalent concomitant diseases. Again, these were statistically higher in the elderly group	Elderly patients can also receive LC without risk. Comorbidity, however, should be kept in mind since it may complicate the procedure and the postoperative recovery period
Loozen et al., 2018 [16]	Netherlands	703	Two groups > 75 years old with 121 patients (17%) and 582 (83%) <75 years old	Acute calculous cholecystitis admitted through the emergency room	- LC: 98% and 99% of patients in the elderly and non-elderly groups. -Primary OC: In both groups, three patients	An ASA score> III was seen statistically significantly more in senior individuals (37% vs. 8%)	Cardiovascular illness (28% vs. 11%), lung disease (23% vs. 7%), and diabetes (19% vs. 11%) were all more prevalent and statistically significant in elderly individuals	Elderly patients with mild to moderate acute cholecystitis respond well to early laparoscopic cholecystectomy
Shin et al., 2018 [17]	Korea	205	Three groups: group A (<65 years old), group B (from 65 to 79 years old), and group C (>79 years old)	AC	-LC: 114 patients in group A, 70 in group B, and 21 in group C	Elderly patients (Groups B and C) had significantly higher ASA scores than group A	The underlying diseases of the patients varied significantly depending on the age group. For example, in group C(>79 years old), diabetes and hypertension were more common	Complication and conversion rates were comparable across age groups when adequate preoperative assessment and therapy were carried out. Therefore, patients over 80 can safely undergo LC if a comprehensive preoperative evaluation of their health and an elective procedure is carried out
Escartín et al., 2019 [18]	Spain	348	Two groups: those under the median age of 85.4 years (Group A) and those over that age (Group B)	AC in patients ≥80 years	-Antibiotics only: 91 patients in Group A and 119 in Group B -LC: 72 patients in Group A and 32 in Group B -OC: 15 patients in Group A and 7 in Group B	Poor physical condition (ASA III-IV), and ASA IV, were associated with an increased risk of serious complications. Forty-three patients were ASA IV, 17 from Group A and 26 from Group B	Diabetes was present in 104 patients (30%); 52 patients were from Group A and 52 from Group B	The severity of the disease (Grade III AC) and/or poor physical condition (ASA III-IV), rather than age per se, limited the therapeutic options available to older AC patients. Even in elderly individuals with Grade I–II AC, LC can be successfully conducted safely. When Grade III AC, there is a substantial risk of morbidity and death, necessitating a customized treatment plan
Oldani et al., 2019 [19]	Italy	245	Two groups: group A (>80 years old) and group B (<80 years old)	AC managed with cholecystectomy during the same hospitalization	-Primary OC: Group A 9 patients (16.1%), and Group B 21 patients (11.1%). -LC: Group A 43 patients (76.8%), and Group B had 153 (81%)	The ASA score indicates that older participants tend to be at higher risk	NR	For patients over 80 years of age and at high risk, minimally invasive therapy for acute cholecystitis appears to be a practical and effective therapeutic option
Coelho et al., 2020 [20]	Brazil	1,645	Two groups: elderly (≥60 years of age) and younger (<60 years of age)	Acute and chronic cholecystitis	- LC: 484 (29.7%) of the patients were older, and 1,161 (70.3%) were younger	In the younger group, there were more patients with ASA I; in the elderly group, there were more patients with ASA II, III, and IV	NR	The elderly can undergo LC with acceptable rates of morbidity and mortality. When elderly patients have LC, it takes longer to complete the procedure, and they are more likely to develop acute cholecystitis, convert to open cholecystectomy, and experience postoperative problems. Age is less of a predictive factor for LC than the severity of gallbladder disease
Serban et al., 2021 [21]	Romania	333	Four subgroups: Group A ≤49 years; Group B: 50–64 years; Group C: 65–79 years; Group D ≥80 years	Acute cholecystitis admitted through the emergency department	-LC: Group A:119 (97.5%) patients; Group B: 99 (89.2%); Group C:51 (77.3%), and Group D:26 (76.5%). -OC: Group A:1 (0.8%) patient; Group B: 4 (3.6%); Group C:5 (7.6%), and Group D:2 (5.9%)	The increase in ASA score with aging was supported by the Linear-by-Linear association test, which revealed that there were substantially fewer patients in groups C and D compared to groups A and B with ASA PS risk I and significantly more patients with ASA PS III	From 2.5% in group A to 44.1% in group D, the incidence of acute cardiac insufficiency at admission significantly increased with age. In addition, similar connections between decompensation in the context of systemic inflammation and sepsis and creatinine levels >1.2 mg/mL, a manifestation of a pre-existing age-related impairment of renal function, were discovered	The critical variable affecting the unfavorable result of LC in the elderly was the level of systemic inflammation. Diabetes was linked to higher surgical and systemic postoperative morbidity among comorbidities, while stroke and chronic renal insufficiency were linked to a higher risk of cardiovascular problems. With proper perioperative care, elderly patients can significantly benefit from a minimally invasive procedure's advantages, which include a lower risk of postoperative complications and a shorter hospital stay

**Table 8:** Main characteristics of the cross-sectional study, case-control study, and systematic review and meta-analysis included in the review NR: not reported; AC: acute cholecystitis; LC: laparoscopic cholecystectomy; OC: open cholecystectomy; ASA PS: American Society of Anesthesiologists Physical Status Classification; CCI: Charlson Comorbidity Index; LOS: length of hospital stay; SR-MA: systematic review and meta-analysis

First author, year	Study type	Country of origin	Patient number	Age ranges	Indications for surgery	Type of treatment	ASA PS	Comorbidity	Results and key points
Bass et al., 2021 [13]	Cross-Sectional	Twenty-five centers from 9 countries: Austria, Italy, Ireland, Romania, Spain, Sweden, Portugal, the UK, and the USA	338	Two groups: the <65 years group and the>65 years group	AC 154 patients (45.6%), gallstone pancreatitis 71 patients (21%), common bile duct stone 61 patients (18%), and cholangitis 47 patients (13.9%)	-LC: 86 patients (86.9%) in the <65 years' group, and 56 patients (80%) in the >65 years' group. OC: Eight patients in the <65 years' group and five in the >65 years' group	According to their ASA scores, patients over 65 were less surgically fit than those under 65	Patients over 65 had more significant comorbidities than those under 65, as determined by their Charlson Comorbidity Index (CCI)	Surgery is rarely used to treat acute complicated calculous biliary disease in older patients. Postoperative morbidity, LOS, and the need for post-discharge rehabilitation are inevitably higher in older patients than younger ones. However, they are otherwise similar to senior patients treated non-operatively. Older patients may receive the best care possible in a secure environment using comprehensive perioperative optimization
Xu et al., 2022 [22]	Case-Control	China	94	Ninety-four elderly patients were assigned to a research group (n=47) and a control group (n=47)	AC	LC was given to the study group within 48 hours of the onset of AC; 48 hours after the start, LC was given to the control group	NR	There was no discernible intergroup variation in the underlying illnesses	Elderly individuals with AC who receive early LC treatment benefit from postoperative functional rehabilitation and experience minimal effects on their energy metabolism. Early LC treatment is superior to LC treatment administered after 48 hours because it causes less severe stress reactions, has lower bilirubin content, less inflammatory response, improves liver function, and has a lower incidence of problems
Kamarajah et al., 2020 [23]	SR-MA	Ninety-nine studies, from North America (n=28), Asia (n=31), and Europe (n=39)	326,517	Age subgroups: 60 years and older, 65 years and older, 70 years and older, 75 years and older, and 80 years and older	Only 77 studies documented surgical indications. In 90 investigations (n=303,463 patients), the urgency of cholecystectomy was recorded, with 7% (n=19,754) elective cases and 67% (n=203,924) emergency cases	-LC in the elderly population	NR	NR	After laparoscopic cholecystectomy, overall serious complications are more common in the elderly. There is a corresponding sevenfold rise in perioperative mortality, which rises to tenfold in patients over 80. This study supports prior assumptions that older patients undergoing cholecystectomy face increased risks and will help with treatment planning and informed consent

The cut-off ages utilized to designate elderly populations varied significantly, with age 60 (n=3 studies), 65 (n=3 studies), 70 (n=1 research), 75 (n=1 study), and 80 (n=3 studies) being the most common. The features and outcomes of the studies were described using the age groups established by each study. There were a total of 375,623 elderly patients in the 11 studies.

The systematic review and meta-analysis was the study with a significant population of 326,517 elderly patients (>60 years old) receiving laparoscopic cholecystectomy. Also, this study mainly emphasized the perioperative results of the surgery. In contrast, the rest of the studies reported the patients' physical status and comorbidities before the surgical intervention.

Outcomes

The authors analyzed all the studies in search of the overall complications reported, the rate of conversion to open surgery, bile leaks and biliary tract injury, postoperative mortality, and length of hospital stay. Unfortunately, some of the secondary results were not reported due to the different methods used in the study design. The resumed studies' outcomes are presented in Table 9.

**Table 9:** Outcomes addressed by the included articles NR: not reported; AC: acute cholecystitis; LC: laparoscopic cholecystectomy; OC: open cholecystectomy; ASA PS: American Society of Anesthesiologists Physical Status Classification; CCI: Charlson Comorbidity Index; ICU: intensive care unit; LOS: length of hospital stay

First author, year	Outcomes addressed				
	Overall complications	Conversion to open surgery	Bile leaks and biliary tract injury	Postoperative mortality	Length of hospital stay
Wiggins et al., 2018 [14]	The overall 30-day mortality was 10.2% (4,829), of which the conservative management cohort had 4,240 deaths, emergency cholecystectomy 409, and percutaneous cholecystostomy 180	NR	Seventy-six patients (2.1%) suffered bile duct injury, and biliary leakage was reported in 47 (1.3% ) patients who received cholecystectomy	Elderly patients undergoing cholecystectomy have a mortality rate of 11.6%	NR
Ekici et al., 2018 [15]	Forty-seven patients (7.1%) experienced postoperative complications, and these were statistically substantially greater in the age group >60 years	LC was Converted to open in 5.6% (37 patients), 12 patients (10.4%) >60 years of age, and 25 patients (4.6%) <60 years of age	Five cases of biliary tract damage were noted (0.8%). Three (2.4%) of these patients were over 60	In the elderly group, 0.8% was estimated as the death rate	Hospitalization duration was statistically higher in the elderly, with a mean of 1,28 days
Loozen et al., 2018 [16]	The perioperative morbidity and mortality rates for elderly patients were 17% and 3%, respectively, but these rates were better for non-elderly patients at 8% and 1%, respectively	Conversion to open cholecystectomy was substantially more prevalent among the elderly (18% vs. 5%)	Three cases had biliary tract damage; one (0.8%) of these patients was over 75. Biliary leakage was reported in 10 patients, the most common complication in the elderly group with eight patients (7%)	Mortality rates for elderly patients were 3% and 1% for the non-elderly group	The Elderly group's median postoperative hospital stay was longer and statistically significant (5.0 vs. 3.0 days)
Shin et al., 2018 [17]	The distribution of surgical complications among the various age groups indicated no variation. Non-surgical complication rates: Group A had a 2.6%, Group B 7.1%, and group C 4.8%. There were no discernible variations across the age groups	Eight of the 114 patients in group A, seven of the 70 in group B, and one of the 20 in group C required open conversion. It was not statistically significant	Common bile duct injury was present in three cases, one patient from each age group. In addition, biliary leakage was reported in two (2.9%) patients of group B	NR	Age-related differences in hospital stay duration suggested that older patients required more extended time for postoperative recovery
Escartín et al., 2019 [18]	Grade I-II AC: No deaths in group A and morbidity was minimal in both operated and non-operated patients (12.5% vs. 8.2%). Group B had one death and more significant complications when they underwent surgery (24% vs. 5.8%). Grade III AC: Severe complications were more frequent in group A patients who underwent surgery than in those who did not (53% versus 50%, respectively), and deaths were high (11.8% in the patients that underwent surgery, compared to 25.0% in those who did not). In group B, the proportion of deaths and severe complications was similar	Conversion to open surgery: A total of 12 (11.5%), nine patients in Group A, and three in Group B	NR	Grade I-II AC: In group A: no fatalities, and in group B: one (4.2%) death. Grade III AC: group A: two (11.8%) deaths and group B: five (27.8%) fatalities in the non-operated patients and three (30%) in the operated patients	Hospital stays in group A: between 5.5 and 8.1 days in non-operated and from 5.3 to 10.4 days in the operated group. In comparison, group B was between 5.6 and 12.1 days in non-operated and from 7.5 to 8.2 days in operated
Oldani et al., 2019 [19]	The mortality (5.4%) and morbidity rate in group A were more significant (14.3% vs. 3.1%), primarily because severe immediate postoperative cardiovascular and respiratory problems occurred more frequently than any other complication unrelated to the surgical procedure	-Conversion to open surgery: A total of 19 cases, four patients (7.1%) from Group A and 15 patients (7.9%) from Group B	Common bile duct injuries were present in two patients of group B	The mortality rate in group A was 5.4%(2 cases of postoperative myocardial infarction and one instance of acute respiratory insufficiency), while no deaths were reported in group B	The two groups had a similar median hospital postoperative stay (five vs. four days), which was not statistically different.
Coelho et al., 2020 [20]	Similar rates of intraoperative complications were observed in both groups, but the rate of postoperative complications was more significant in the older group (7.2% vs. 4.7%) than in the younger group. Postoperative mortality was significant in the elderly too	Compared to the younger group, the conversion rate for open cholecystectomy was higher in the elderly (0.8% vs. 0.09%)	NR	Elderly patients had three (0.6%) postoperative deaths; the younger patients did not experience any fatalities	The length of hospital stays in the groups was the same
Serban et al., 2021 [21]	Patients above 50 had a considerably higher rate of postoperative complications (p=0.045). Also, Age-related increases in the frequency of postoperative severe cardiovascular complications were seen. However, the incidence of postoperative complications and the prevalence of cardiovascular problems in groups B, C, and D did not differ statistically significantly	Patients over 50 had much higher conversion rates than younger patients (Group A:1,6%, Group B:7.2%, Group C:15.2%, and Group D:17.6%)	One main bile duct injury was reported	Mortality reported: Group A: one patient (0.81%); Group B: two patients (1.8%); Group C: One patient (1.51%); and Group D: no fatalities	Laparoscopic cholecystectomy results better than open cholecystectomy in shorter postoperative and hospital stays
Bass et al., 2021 [13]	Patients over 65 reported greater postoperative complications than younger patients. Statistically, the difference was not significant. (18.6% vs. 10.1%, p=0.114). Nineteen patients (5.6%) were transferred to the ICU during their hospital stay due to organ failure, with 10 (6.5%) of them being under the age of 65 and nine (4.8%) being over that age	-Conversion to OC: five patients (5.0%) in the <65 years' group and nine patients (12.9%) in the >65 years' group	Bile duct complications were reported in three patients (3.0%) under 65 and six (8.6%) over 65 years	Patients over 65 experienced four deaths (2.2%) compared to one patient under 65 (0.7%). In addition, patients who passed away had a heavier burden of comorbidities	Postoperative (days median 5.0 vs. 3.0 ) and overall hospital length of stay (days median 7.5 vs. 7.0) durations were significantly longer among individuals over 65
Xu et al., 2022 [22]	The research group experienced fewer complications (4.26%) than the control group (17.02%); this difference was statistically significant	There was no conversion rate reported	Biliary leakage was reported in two (4.26%) control group patients	There was no reported mortality	The research group's hospital stay was shorter than the control group's. (median of 4.46 vs. 6.73 days)
Kamarajah et al., 2020 [23]	Forty-eight studies involving 49,215 participants reported overall complications. Overall complication rates were significantly higher in the age subgroups: 65 years and older. When stratified by urgency, older patients had significantly more overall complications for elective and emergency procedures	In 53 studies with 59,173 patients, the effect of advancing age on conversion to open cholecystectomy was documented. The likelihood of conversion to open cholecystectomy was substantially higher in subgroups aged 65 and older	In 30 studies involving 42,765 patients, the effect of aging on bile leakage was documented. Overall, there was a significant correlation between age and bile leakage	Fifty studies with 78,404 patients reported the effect of aging on postoperative mortality. All age cut-off groupings showed a substantial rise in postoperative death rates	In 24 research involving 10,997 patients, the effect of advancing age on the post-cholecystectomy duration of stay was documented. Overall, a more extended stay was substantially correlated with older age

Discussion

AC in the elderly is becoming more prevalent as the population ages. However, while patients' general health improves as they age, having comorbid conditions makes choosing a treatment more difficult. This systematic study evaluates the safety of minimally invasive cholecystectomy and opens cholecystectomy in this population compared to younger patients.

The likelihood of a patient undergoing a cholecystectomy after presenting with symptomatic gallstone disease as AC decreases with age [25]. This was shown by Wiggins et al., where only 7.5% of the elderly patients had a cholecystectomy, and the majority of patients (89.7%) had conservative treatment [14]. Also, Escartín et al. reported increased use of conservative treatment with increased age [18]. However, the rest of the studies did not note differences in the treatment received.

When comparing the type of surgery, half of the studies reported a laparoscopic procedure as the preferred course of treatment [15,17,20,22,23]. In contrast, in the rest of the studies, the number of open approaches was minimum. Due to this, a proper comparison between the open and laparoscopic procedures could not be made because the open procedure was not a first-line surgical treatment option regardless of the patient's age [13,16,18,19,21]. Furthermore, none of the studies uses robotic-assisted cholecystectomy.

In the case of preoperative physical status (ASA), elderly patients have higher score distribution; this was reported by Serban et al. and supported by the linear-by-linear association test [21]. Furthermore, ASA III and higher were more significant in the elderly group in most studies, showing an increased surgical risk. However, according to the same research, surgery is safe if an adequate preoperative assessment is performed on the elderly [13,15-21].

Elderly patients have a higher burden of comorbidity when compared to their younger counterparts, which results in a higher frequency of complications [26]. This is evidenced by a significantly higher CCI in elderly groups [13,14]. Cardiovascular illness, lung disease, and diabetes were the most prevalent concomitant diseases [15-18]. Consequently, Wiggins et al. reported fewer comorbidities in patients who underwent emergency cholecystectomy [14].

Overall Complications

In general, an increase in age has been substantially linked to higher risks of surgical complications. Most of the studies in this review reported an increase in overall complications associated with the increasing age of the patients. However, the age of cut-off varies in the studies. For example, Serban et al. reported an increased rate of postoperative complications in patients over 50 and an increase in age-related cardiovascular postoperative complications [21]. In the same way, Bass et al. and Kamarajah et al. reported higher postoperative problems in patients over 65 [13,23]. However, Escartín et al. report increased complications with the increase in AC severity. Serious complications are more frequent in patients with grade III AC, independent of the treatment [18]. Also, Loozen et al. discovered that severe AC was linked to higher comorbidity [16].

Conversion to Open Surgery

The probability of converting to an open cholecystectomy rose considerably with age. This was consistently reported by the majority of the studies [13,15,16,19,20]. These results contrast with the study conducted by Shin et al., where no significant differences in conversion rate were discovered [17]. Also, the effect of advancing age on conversion to open cholecystectomy was documented in 53 studies with 59,173 patients, forming part of the systematic review and meta-analysis by Kamarajah et al. [23]. This result is consistent with the literature, which shows that advanced age increases the risk of converting to an open procedure [27].

Bile Leaks and Biliary Tract Injury

There was a significant correlation between age and bile leakage [13,16,19]. Similarly, Kamarajah et al. reported that the effect of aging on bile leakage was documented in 30 studies involving 42,765 patients [23]. In the same way, biliary tract injury was reported more frequently in elderly patients [15,16]. However, Shin et al. did not find a difference in the incidence of biliary tract injury between old and young groups [17].

Postoperative Mortality

Aging was substantially linked to higher postoperative mortality rates [13,15,16,18-20]. Likewise, Kamarajah et al. reported that the effect of aging on postoperative mortality was observed in 50 studies with 78,404 patients [23]. Moreover, Wiggins et al. stated that in the case of elderly patients undergoing emergency cholecystectomy, the mortality rate can be as high as 11.6%. However, one of the major flaws of this research was that it did not consider the associated comorbidities and their impact on the outcomes [14]. In contrast with these findings, Antoniou et al. discovered that mortality was 1.0% for the laparoscopic approach and 4.4% for the open approach in a meta-analysis of 11 studies published between 1993 and 2011 involving 101,559 patients aged 65 years or older (48,195 treated laparoscopically and 53,364 by open cholecystectomy) [28].

Length of Hospital Stay

In most studies, a more extended hospital stay was substantially correlated with older age [13,15-18,23]. However, Coelho et al. reported no difference in the length of hospital stays between the elderly and the younger group [20]. On the other hand, Serban et al. expressed that laparoscopic cholecystectomy produces better results than open cholecystectomy in terms of shorter postoperative and overall hospital stays [21], whereas Xu et al. showed that early LC in elderly patients with AC leads to shorter hospital stays after surgery [22].

Overall Outcomes

In elderly patients with mild to moderate AC, minimally invasive therapy appears to be a practical and effective therapeutic option. Conservative treatment is deemed ineffective based on existing research [14,18,29,30]. Comorbidities, however, should be kept in mind since they may complicate the procedure and the postoperative recovery period [15,16,18,19,21]. Complication and conversion rates are comparable across age groups when adequate preoperative assessment and therapy are carried out [17]. The severity of the disease (grade III AC), poor physical condition, and/or comorbidities rather than age per se can limit the therapeutic options available to older AC patients [13,18,20,21]. Also, reduced readmission rates and one-year mortality are two potential advantages of emergency cholecystectomy in very elderly patients reported [14]. However, Kamarajah et al., in their meta-analysis of 99 studies, support prior assumptions that older patients undergoing cholecystectomy face increased risks and reported a seven-fold rise in perioperative mortality, which rises to 10-fold in patients over the age of 80 years. Furthermore, they recommended surgery selection on a patient-by-patient basis [23].

Limitations

This review restricted the included studies to those in the English language and those with a free full text published in three databases between 2018 and 2022. Gray literature and other databases were not used. In addition, the majority of the studies that the search yielded were cohort studies. Furthermore, the review was limited by the heterogeneity of the studies. For example, the studies vary in patient age, preoperative evaluations, and treatment options. No randomized controlled clinical trials (RCTs) or studies involving robotic-assisted cholecystectomy in the elderly with AC were found.

There was no extensive follow-up in the patients; most of the studies focused on the immediate postoperative results instead of preoperative management. Therefore, we recommend observational studies with longer follow-ups after surgery and adequate preoperative preparations as well as RCTs to find out which procedures or treatments provide the most significant benefits to elderly patients.

## Conclusions

The studies included in this review demonstrate that in the elderly, compared with younger patients, the surgical treatment of AC is a challenging decision. The severity of this condition, the diminished physical status, and multiple comorbidities increase the risk of operative and postoperative complications in the older group of patients. Even though there is no consensus on the surgical treatment of AC in elderly patients, the laparoscopic approach is the preferred procedure for cholecystectomies. It can be safely performed in older patients and the conservative approach is not recommended.

The overall complications, open surgery conversions, biliary tract injury, leaks, postoperative mortality, and hospital length of stay increase considerably with age. Nevertheless, minimally invasive cholecystectomy is a feasible treatment option for elderly patients suffering from mild to moderate AC when a comprehensive perioperative assessment is conducted. Most complications reported in old patients who underwent cholecystectomy were related to the burden of comorbidities and cholecystitis severity than to the age or surgical procedure. That being the case, a thorough optimization of elderly patients with severe cholecystitis or severe comorbidities is required to determine the optimal treatment. Furthermore, extensive observational studies and RCTs need to be conducted and guidelines regarding associated diseases, physiological status, and age have to be devised and published.
